# Supplementary material for: Curcumin Derivative GT863 Inhibits Amyloid-Beta Production via Inhibition of Protein N-Glycosylation
Source: Cells. 2020 Feb 3;9(2):349. doi: 10.3390/cells9020349 (PMC7072163; doi:10.3390/cells9020349)
Supplement: Supplementary file 1 [file cells-09-00349-s001.pdf]

Supplementary Figure S1

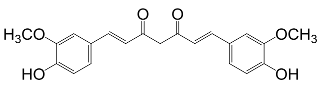

curcumin

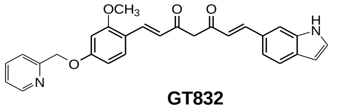

GT832

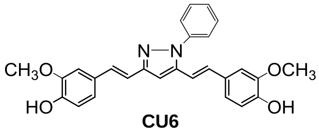

CU6

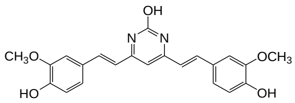

GT934

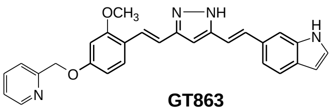

GT863

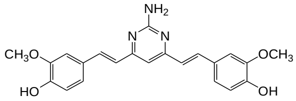

GT935

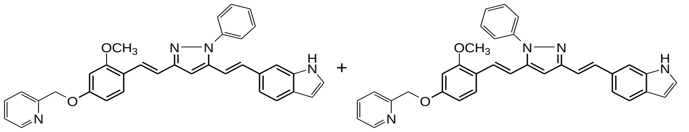

GT855

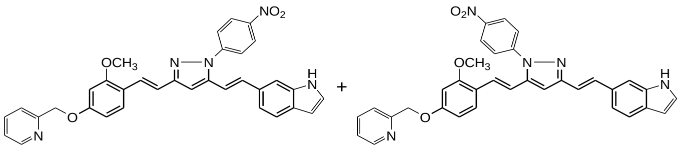

GT857

Supplementary Figure S1
Chemical structural of curcumin derivatives

Supplementary Figure S2

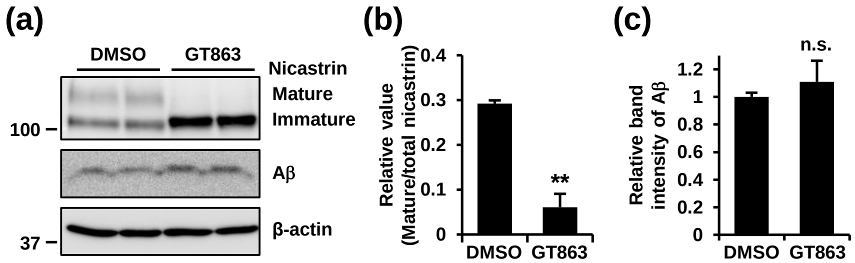

Supplementary Figure S2 Effect of kifunensine treatment on Aβ production in CHO-APPswe cells. (**a-d**) CHO-APPswe cells were treated with 1 μg/ml kifunensine (Kif) for 48 h. (**a**) Whole cell lysates were immunoblotted with appropriate antibodies as indicated. (**b-c**) Band intensities of nicastrin (**b**) and Aβ (**c**) were quantified by densitometric scanning, relative value being shown. mean ± S.D. n = 3, **, $p < 0.01$, n.s. = not significant.
